# The Gut Microbiome and Colorectal Cancer: An Integrative Review of the Underlying Mechanisms

**DOI:** 10.1007/s12013-025-01683-9

**Published:** 2025-02-13

**Authors:** Farah Karam, Yara El Deghel, Rabah Iratni, Ali H. Dakroub, Ali H. Eid

**Affiliations:** 1 Faculty of Medicine, University of Balamand, Al-Koura, Lebanon; 2 Department of Biology, College of Science, United Arab Emirates University, Al-Ain, UAE; 3 Blavatnik Family Research Institute, Departments of Cardiology and Population Health Science and Policy, Icahn School of Medicine at Mount Sinai, New York, NY USA; 4 Department of Basic Medical Sciences, College of Medicine, QU Health, Qatar University, Doha, P.O. Box 2713, Qatar

**Keywords:** Colorectal cancer, Microbiota, Gut, Tumor, Bacteria, Probiotics, Dysbiosis

## Abstract

Colorectal cancer (CRC) is the second leading cause of cancer-related mortality worldwide. While the incidence and mortality of CRC have decreased overall due to better screening, rates in adults under 50 have risen. CRC can manifest as inherited syndromes (10%), familial clustering (20%), or sporadic forms (70%). The gut microbiota, comprising mainly firmicutes and bacteroidetes, play a key role in CRC development and prevention. Indeed, CRC progression is influenced by the dynamic interaction between the gut microbiota, the intestinal barrier, the immune system, and the production of short-chain fatty acids. Not surprisingly, imbalance in the gut microbiota, termed dysbiosis, has been linked to CRC due to ensuing chronic inflammation, DNA damage, and oxidative stress. This may explain the notion that probiotics and fecal microbiota transplantation offer potential strategies for CRC prevention and treatment by restoring microbial balance and enhancing anti-cancer immune responses. This review appraises the roles of gut microbiota in promoting or preventing CRC. It also discusses the mechanistic interplay between microbiota composition, the intestinal barrier, and the immune system, with the hope of developing potential therapeutic strategies.

**Graphical Abstract:**

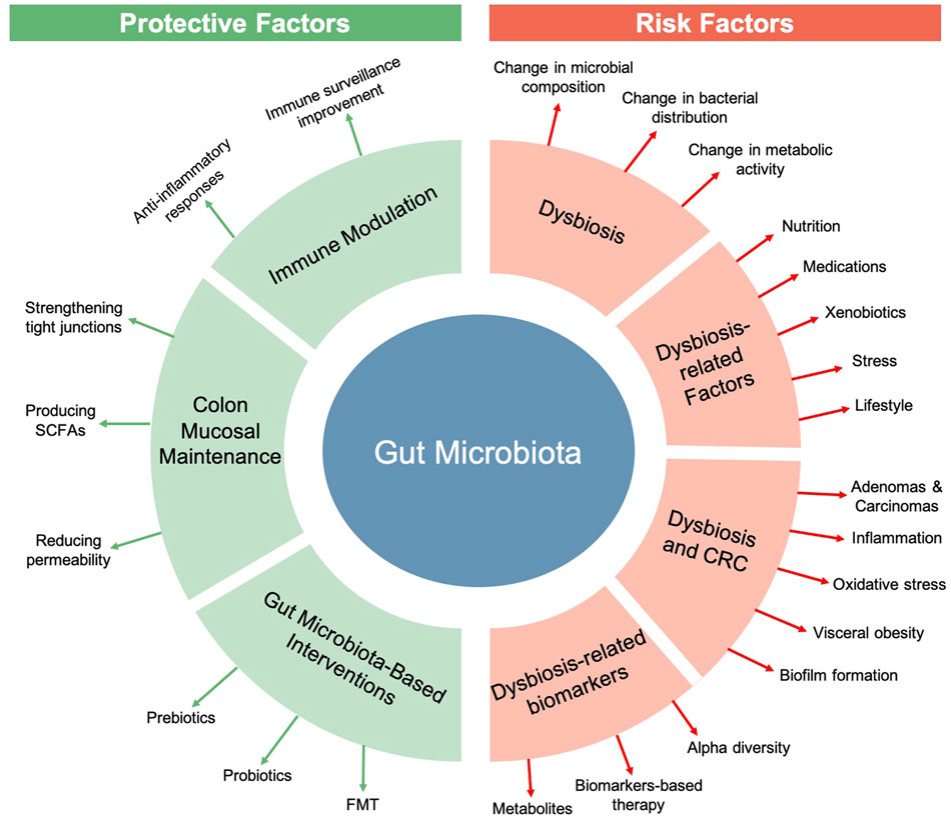

## Introduction

Colorectal Cancer (CRC) is a gastrointestinal cancer that originates from the colon or rectum [1]. Colon cancer (CC) and rectum cancer (RC) develop in the large intestine and are usually referred to as a single tumor entity (CRC) [2]. CRC is the second most common cause of death and the third most frequent type of malignancy for both sexes combined [2]. The incidence of new cases and deaths has been gradually decreasing, except for adults younger than 50 years old [3]. A noticeable trend of younger individuals being diagnosed at more advanced stages, in the left colon or rectum, has been observed over the past years [4].

CRC can present as an inherited syndrome (10%), familial clustering (20%), or, in most cases, as sporadic (70%) [5]. Inherited CRC is divided into hereditary polyposis CRC and hereditary non-polyposis CRC [6]. The hereditary polyposis form is further divided into the Familial Adenomatous Polyposis (FAP) and attenuated polyposis. FAP is characterized by the development of hundreds to thousands of polyps in the colon and rectum, which if left untreated almost always lead to cancer. The condition is caused by mutations in the *APC* gene, a tumor suppressor that normally helps regulate cell growth. The progression to cancer involves a series of genetic alterations, known as the chromosomal instability pathway. This includes mutations in the *KRAS* gene, which regulates cellular signaling, and mutations in the *TP53* gene, another tumor suppressor gene. The accumulation of these mutations promotes tumorigenesis [7]. In contrast, hereditary non-polyposis CRC, also known as the Lynch syndrome, is the most common form of hereditary CRC [8]. Lynch syndrome results from mutations in mismatch repair genes, such as *MLH1, MSH2, MSH6, and PMS2*, which are responsible for repairing DNA replication errors [6, 8]. Mutations in mismatch repair genes lead to microsatellite instability (MSI), where the length of microsatellite DNA repeats varies due to unrepaired insertion or deletion errors [8]. Both types of hereditary CRC follow the classic or traditional tumor model. According to the traditional model, cancer starts with an aberrant crypt focus that progresses to the formation of a benign adenomatous polyp. This polyp evolves into an early tubular or tubulovillous adenoma and later progresses to CRC [9]. The familial clustering form is characterized by the absence of identifiable inherited syndromes while still having a familial disposition [10]. The serrated polyposis syndrome appears to have a familial component, but the genetic core remains unknown [11]. It is characterized by hyperplastic polyps which originate from the sessile serrated polyps and constitute around 5 to 10% of polyps [9].

Most cases of CRC are rather sporadic. The chromosomal instability pathway is the main driver of sporadic CRC and includes *APC*, *KRAS*, and *TP53* mutations [12]. Other factors can promote the development of sporadic CRC. For instance, age is a major risk factor for sporadic CRC. The average age at diagnosis is 50 years, but it occurs at much younger ages in patients with hereditary CRC [3]. Moreover, non-cancerous diseases such as colorectal polyps, adenomas, ulcerative colitis, and Crohn’s disease increase the risk of developing CRC. In addition, environmental determinants such as lifestyle, diet, and the microbial community can trigger tumorigenesis [13]. The gut microbiota has been shown to play a dual role in CRC, both protecting against it and, under certain conditions, promoting its development [13].

## Gut Microbiota

Gut microbiota compromises microorganisms occupying the human gastrointestinal tract [14]. It is a complex, vigorous, dynamic, and diverse ecosystem of microbes that interact with each other and the human host [15]. Gut microbiota consists mainly of prokaryotic organisms (bacteria and other unicellular microbes), in addition to viruses, fungi, parasites, and archaea. The genetic profile of the microbiota is called the microbiome and consists of over 3.3 million genes compared to the human genome of approximately 23,000 genes [16]. The gut microbiota mainly consists of bacteria, with a few phyla including up to 160 species. The two main phyla: the Firmicutes and the Bacteriodetes constitute 90% of the gut microbiota. Other phyla include Fusobacteria, Proteobacteria, Verrucomicrobia, and Actinobacteria. Among the main phyla, the Firmicutes phylum embraces more than 200 genera including *Bacillus*, *Enterococcus*, *Lactobacillis*, *Clostridium* (95%), and *Ruminicoccus*. On the other hand, the Bacteriodete phylum is mainly composed of the *Bacteroides* and the *Prevotella* genera [17].

The composition of the human microbiota is diverse and unique among individuals. The influence of genetics on gut microbiota composition is limited compared to environmental factors such as diet, lifestyle, and exposure to different microbial communities [18]. Human gut microbiota is seeded before birth [19]. Maternal microbiota builds the first microbial inoculum that diversifies rapidly after birth to form the adult-like gut microbiota by the end of the first 3–5 years of life. Prenatal factors including the mode of delivery (vaginal birth vs. cesarean section), diet (breastfeeding vs. formula feeding), genetics, and intestinal mucin glycosylation have been shown to influence microbial colonization in the newborn’s gut [19]. An estimation of 10^13^ to 10^14^ microbes/mL of 500–1000 species colonize the GI tract within the first 3–5 years of life. After that, a microbial signature is set for each individual which is thought to influence the overall health of the human body host [16, 18].

## Gut Microbiota as a Protective Factor for Colon Cancer

### Colon Barrier Structure and Functions

The intestinal barrier constitutes an essential semipermeable barrier of the GI tract, forming an interface between the external environment and the internal milieu of the body [20]. The intestinal barrier is composed of three main layers. The outermost layer includes the mucus layer, the gut microbiota, and defense proteins. The middle layer is formed by the intestinal epithelial cells. Finally, the innermost layer where the contains immune cells [21, 22].

The outermost layer is the first to encounter ingested external molecules as a primary line of defense. It primarily consists of a mucous layer rich in highly glycosylated mucin proteins with mucin 2 (*MUC2*) being the most abundant. *MUC 2* is secreted by specialized epithelial cells in the intestines known as goblet cells [23]. The mucous layer acts as a physical barrier that protects the underlying epithelial cells from direct contact with the gut microbiota and environmental bacteria [23]. In contrast to the single mucus gel layer in the small intestine, the colon has a more complex mucus structure, consisting of two distinct layers: an inner dense mucus layer that is nearly devoid of bacteria and an outer loose mucus layer with a high concentration of commensal bacteria. This structural difference maintains a balance between preventing bacterial invasion and allowing beneficial bacteria to reside close to the intestinal surface. The mucus layer contains antimicrobial peptides and other defense proteins that help control the gut microbiota composition. These proteins, along with the resident gut microbiota, play a role in pathogen exclusion and immune modulation [24, 25].

The intermediate layer of the intestinal barrier is the epithelial cells layer. It consists of five intestinal epithelial cell (IEC) types: enterocytes, Paneth cells, enteroendocrine cells, goblet cells, and microfold cells. Enterocytes are the most abundant cells and are primarily responsible for nutrient absorption. Paneth cells are located at the base of the crypts in the small intestine and secrete antimicrobial peptides to help maintain the intestinal flora. Enteroendocrine cells secrete hormones that regulate various physiological processes, including digestion and appetite. Goblet cells secrete mucous, contributing to the protective mucus layer. Microfold (M) cells are specialized for antigen sampling and play a role in immune surveillance [26]. This epithelial layer is impermeable to hydrophilic solutes making molecules and nutrients pass via transporter proteins [21]. In addition, the protective lining restricts the entry of toxins and microorganisms. The epithelial layer is formed by a barrier of the three junctions. From apical to basal, the epithelial layer includes tight junctions (TJs) or zonula occludens, adherence junctions (AJs) or zonula adherence, and desmosomes or macula adherens. The TJs are responsible for blocking microbial invasion by forming links between epithelial cells. Those junctions also seal the paracellular spaces between the cells allowing the selective absorption of nutrients and ions. On the other hand, the AJs provide mechanical adhesion between epithelial cells, maintaining the integrity of the epithelial layer. Desmosomes contribute to the mechanical strength of the epithelium and help it withstand physical stress [27, 28].

The innermost layer of the intestinal barrier contains various immune cells. This includes epithelial cells, intraepithelial lymphocytes (IELs), Paneth cells, cells of innate and adaptive immunity, and gut-associated lymphoid tissue (GALT). Epithelial cells form a physical barrier and play an active role in immune responses by sensing and signaling the presence of pathogens. Intraepithelial lymphocytes (IEL) are immune cells located within the epithelial layer that mediate immediate immune responses to pathogens. Moreover, Paneth cells, besides their role in antimicrobial peptide secretion, contribute to the regulation of the gut microbiota and support epithelial cell turnover. Cells of the innate and adaptive immune systems, including macrophages, dendritic cells, T cells, and B cells, work together to identify and respond to pathogens. In addition, GALT, which includes Peyer’s patches and isolated lymphoid follicles, facilitates antigen sampling and initiates immune responses [21, 29, 30].

### Gut Microbiota and Colon Barrier

The microbiome regulates intestinal homeostasis through a mutualistic relationship with the host [27, 31]. Host health has been closely linked to the integrity of the GI tract barrier, which is partly maintained by the microbiome residing in the outermost layer [22, 27]. Gut bacteria maintain intestinal homeostasis via multiple mechanisms. Microbiota regulates epithelial permeability and integrity [32, 33]. The gut bacteria-epithelial cell interactions regulate permeability by modulating tight junctions [33]. For instance, the human symbiont *Lactobacillus reuteri* increases the expression of tight junctions in the epithelial cells and enhances the mucosal barrier by reducing permeability dysfunction [34]. Due to its potency, this bacterium has been used as a probiotic in the treatment of multiple intestinal diseases [34]. A common feature of CRC is gut barrier dysfunction and increased permeability [35]. *L. plantarum, L. rhamnosus, and E. coli Nissle 1917* act as potent probiotics that normalize or upregulate the expression of tight junctions, including claudin-1, occlude, ZO-1, and ZO-2 [36]. These, along with other gut bacteria produce short-chain fatty acids (SCAFs), such as butyrate, during the fermentation of dietary fibers. Butyrate is a major energy source for colonic epithelial cells and has been shown to enhance the expression of tight junction proteins [37].

Gut microbiota is necessary for the appropriate function and integrity of the intestinal mucus layers. During intestinal homeostasis, mucin-utilizing bacteria are required for the adequate turnover of the mucin protein to ensure an effective barrier [32]. Rats having a thin or even absent mucus layer have been found to be germ-free (GF) rats [24]. In addition, histochemical analysis revealed a thin colonic mucus layer in GF rats compared to conventionally raised rats. Similar results were shown in GF adults compared to raised rodents. This suggests that the presence of gut microbiota is necessary for the adequate production and maintenance of mucus. In newborns, the expression of mucin genes (*Muc1-4*) is minimal due to the initially low levels of gut microbiota [24]. Mucin glycans, the carbohydrate components of mucins, serve as nutritional sources for intestinal bacteria, which enable them to thrive when diet-derived glycans are limited [38].

### Gut Microbiota and The Immune System

**Fig. 1:**
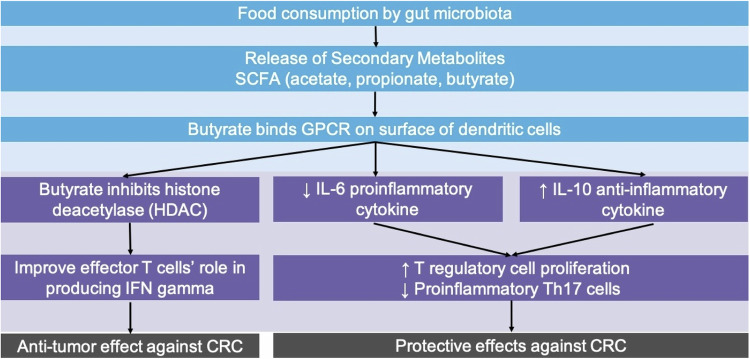
Gut microbiota Modulation of the host’s immunity against Colorectal Cancer. Short-chain fatty acids (SCFAs) such as acetate, propionate, and butyrate are secondary metabolites released by gut microbiota following food consumption. Butyrate binds to G-protein coupled receptors (GPCR) on the surface of dendritic cells, leading to several immune-modulatory effects. These include a decrease in the proinflammatory cytokine IL-6 and an increase in the anti-inflammatory cytokine IL-10. Butyrate promotes the proliferation of regulatory T cells while decreasing proinflammatory Th17 cells, contributing to protective effects against colorectal cancer (CRC). Butyrate also acts as a histone deacetylase (HDAC) inhibitor to enhance the function of effector T cells in producing IFN-gamma and contributes to the anti-tumor effects against CRC

Gut microbiota interacts with and protects host’s immunity during all stages of CRC [39, 40]. The influence of gut microbiota on CRC has been demonstrated through the digestion of dietary components, modulation of the tumor microenvironment, and antigen mimicry [39, 41, 42]. The gut microbiota utilizes digested dietary components as a tool to modulate the host’s immunity against CRC (Fig. 1). During digestion, gut bacteria ferment dietary fibers to produce secondary metabolites, such as short-chain fatty acids (SCFAs), which are key players in the interaction between microbiota and host immunity [40]. SCFAs, including acetate, propionate, and butyrate bind to G-protein coupled receptors (GPCRs) present on the surface of immune cells, such as dendritic cells [43, 44]. These interactions result in decreased production of proinflammatory cytokines like interleukin-6 (IL-6) and increased production of anti-inflammatory cytokines like IL-10. This cytokine profile supports the proliferation of regulatory T (Treg) cells and suppresses proinflammatory Th17 cells, contributing to a protective effect against CRC [44]. Moreover, butyrate, a main SCFA, is also a histone deacetylase (HDAC) inhibitor. This property allows butyrate to exert anti-tumor effects by promoting apoptosis in cancer cells and enhancing the production of interferon-gamma (IFNγ) by effector T cells, which plays a role in anti-tumor immunity [45, 46].

Microbiota can also protect against CRC by modeling the tumor’s microenvironment, upregulating tumors’ programmed cell death, and degrading toxic tumor products [47]. The tumor microenvironment (TME) is composed of immune cells, cytokines, and effector molecules surrounding the tumor [41]. Gut microbiota has been shown to increase major histocompatibility complex (MHC) class I expression on the surface of dendritic cells facilitating the presentation of tumor antigens and promoting immune detection and clearance of tumor cells [48]. While the expression of programmed cell death-ligand 1 (PD-L1) can be modulated by gut microbiota, the specific effects on immune tolerance and tumor immune evasion are complex and context-dependent [41, 48, 49]. Gut microbiota also works on degrading tumor-released products such as hemolytic glycerophospholipids [45].

Through antigen mimicry, the gut microbiota utilizes the host’s immunity as a protective factor against CRC. Commensal microbiota can work on training the immune system to be prepared for attacking tumor cells by antigen mimicry [50]. Some microbial antigens share common epitopes with tumor-associated antigens, allowing for cross-reactive immune responses. This phenomenon can lead to the priming of the immune system against tumor cells, thereby providing protective effects [42].

### Gut Microbiota-Based Interventions

Probiotics are live microorganisms that regulate microbiota composition when administered in adequate amounts [51, 52]. Those microorganisms are mainly composed of bacteria and yeast, similar to the gut microbiota, and can be administered as supplements or as part of food [53]. Although there are various compositions of probiotics, the main bacteria comprise lactic acid bacteria such as *Lactobacillus* and *Bifidobacterium* species [54]. Other bacteria include *Escherichia*, *Enterococcus*, *Streptococcus*, and *Bacillus*. On the other hand, *Saccharomyces* are the main fungal strains used in probiotics [55]. Probiotics colonize the gut and yield a multitude of health benefits by promoting microbiota composition [51].

Probiotics administration plays an important role in CRC prevention and therapy through several mechanisms [56]. The effects of probiotics can be strain-specific. Probiotics compete against bacterial strains that are well-known to be procarcinogenic such as *Helicobacter pylori, B. fragilis, Salmonella*, and *F. nucleatum*. These pathogenic strains impact gut homeostasis by releasing toxic compounds that disrupt the gut’s epithelium. Probiotics work by inhibiting the proliferation of pathogenic bacterial species, releasing bacteriocins, and reducing pro-carcinogenic enzymes [57]. In addition, probiotics may restrict access of pathogenic bacteria to nutrients, stimulate epithelial barrier function, and compete with pathogenic bacteria at binding sites. The probiotic *E. coli* Nissle 1917 (EcN) can inhibit the enterohemorrhagic E. coli through competitive exclusion of pathogens. The EcN bacterium was shown to secrete a bifunctional periplasmic protein DegP to compete with pathogenic biofilms during dual-species biofilm formation [51]. *E. coli* Nissle 1917 has been used to deliver matrix-tethered therapeutic domains to the gut [58].

Probiotics have been shown to regulate cellular proliferation. For instance, probiotics can induce apoptosis as a preventive mechanism against CRC [59]. The probiotic-derived molecule ferrichrome is a tumor suppressor that can inhibit colon cancer progression [60]. The growth of colon cancer cells is inhibited in conditioned media of *Lactobacillus casei* ATCC334 through secretion of tumor suppressive molecules [61]. The latter was identified to be ferrichrome which induces apoptosis in cancer but not normal healthy cells. This induced apoptosis and inhibition of colon cancer cell growth progression appear to be mediated by JNK, a MAP-kinase that is involved in the regulation of cellular proliferation, differentiation, and apoptosis [61, 62]. Furthermore, *Lactobacillus rhamnosus* has been found to be a probiotic bacterium that exerts a cytotoxic effect and anti-cancer effect in human colorectal adenocarcinoma HT-29 cells [63]. *L. rhamnosus* increases the cell cycle arrest at the G1/G0 phase to prevent cellular proliferation. This bacterial probiotic can enhance the expression of pro-apoptotic genes like *Bax* which subsequently activates intrinsic mitochondrial pathways. This results in an increase in Caspases 3 and 9, release of cytochromes, and induction of apoptosis. In contrast, anti-apoptotic genes such as *Bcl2* are decreased [63].

Fecal microbiota transplantation (FMT) is another potential tool for the prevention or treatment of colon cancer. FMT is the transfer of fecal material from a healthy donor to a patient recipient through the upper or lower gastrointestinal route [64]. FMT is emerging as a promising therapeutic tool due to its potential to reverse microbiota dysbiosis, which is linked to an increased risk of malignancies [65]. FMT has been shown to reverse microbial dysbiosis and upregulate anti-cancer immune responses in CRC mice through different mechanisms [66]. FMT recipients showed a massive infiltration of immune cells including CD8^+^ T and CD49b^+^ NK cells which directly targeted cancerous cells [66]. This effect is thought to be mediated by *Lactobacillus* bacteria [67]. In addition, FMT was shown to trigger an increase of the Foxp3^+^ Treg cells [66] which is thought to be mediated by *Bacteroides fragilis* [68]. Furthermore, cytokines such as IL-1α, IL-6, IL-12α, IL-12β, and IL-17α were reduced, while IL-10 levels were increased [66]. Anti-cancer efficacy was further promoted by the repression of transforming growth factor-beta (TGF-β) and STAT3, and the elevated expression of TNFα, IFNγ, and CXCR4 [66].

Although FMT use in the setting of colorectal cancer remains limited, FMT has been used as a clinical treatment for conditions that precede CRC [65]. For instance, FMT has been used in cases of intestinal bacterial dysbiosis including antibiotic-induced enteritis such as an infection by *Clostridium difficile*, IBD, and IBS [69]. Moreover, FMT has the potential to induce clinical remission in cases of ulcerative colitis [70]. FMT has been also shown to have a promising therapeutic role in the short-term treatment of Crohn’s disease [71]. The use of FMT is also being investigated for other gastrointestinal cancers [72] as well as improving the outcomes of anti-cancer treatments such as chemotherapy, radiotherapy, and immunotherapy [73].

## The Gut Microbiota as a Risk factor for Colon Cancer

### Dysbiosis

Dysbiosis refers to the disturbance of gut microbiota homeostasis, characterized by changes in microbial composition, bacterial distribution, or metabolic activity [74]. A multitude of dysbiosis indexes have been proposed, as dysbiosis remains a poorly defined condition. These indexes have been grouped into five categories: large-scale bacterial marker profiling, relevant taxon-based methods, neighborhood classification, random forest prediction, and combined alpha-beta diversity. Large-scale bacterial marker profiling involves identifying and quantifying specific bacterial markers across large populations to identify common patterns of dysbiosis. The relevant taxon-based methods focus on specific taxa within the microbiota that are known to be indicative of health or disease states. The neighborhood classification classifies microbial communities based on their compositional similarities to known healthy or dysbiotic communities. The random forest prediction is a machine learning approach that uses various microbial and host factors to predict the presence and severity of dysbiosis. In addition, the combined alpha-beta diversity method assesses the overall diversity of the microbiota within an individual (alpha diversity) and between different individuals or groups (beta diversity) [75]. Microbial dysbiosis has been associated with multiple pathological conditions including IBD, diabetes types 1 and 2, autism, obesity, colorectal cancer, and more [76]. Dysbiosis is multifactorial and involved in diverse physiological mechanisms [77].

### Factors Contributing to Dysbiosis

Dysbiosis can be caused by environmental factors such as nutrition, medications, and xenobiotics, or by host-related factors like genetics, health status, stress, and lifestyle [77]. A healthy diet enhances the gut microbiota, strengthens gut barrier function, and leads to the release of anti-inflammatory biomarkers. In contrast, poor and unhealthy diets have been related to microbial dysregulations, metabolic syndromes, compromised intestinal barrier, and systemic inflammation [78]. Furthermore, the use of food preservatives has been associated with gut microbiota dysbiosis. For instance, the addition of antimicrobial food additives leads to microbial dysbiosis caused by the increase in *Proteobacteria* phylum and decrease in *Clostridiales* [79]. Moreover, Nucleotide-binding oligomerization domain-2 (Nod-2) deficient patients were more susceptible to *Proteobacteria* dysbiosis [79]. NOD2 protein plays a role in the immune system’s recognition of bacterial components, and its deficiency has been associated with impaired immune responses, predisposing individuals to inflammation and dysbiosis [80].

Xenobiotics such as antibiotics, heavy metals, and pesticides can alter gut microbiota causing antibiotic-induced dysbiosis. Antibiotics that may induce dysbiosis include streptomycin, vancomycin, metronidazole, and ampicillin [81]. For example, vancomycin can reduce microbial diversity and disrupt the gut ecosystem by targeting gram-positive bacteria, thereby causing a compensatory increase in gram-negative bacteria [82]. The disruption of microbiota due to antibiotics increases susceptibility to diseases; for instance, the administration of clindamycin predisposes to *C. difficile* infection [83]. “Resiliency”, refers to the ability of gut microbiota to recover after antibiotic treatment. Some microbiota communities may recover and return to a baseline state, while others might experience long-term changes or fail to fully recover. This variability in resilience can impact gut health and contribute to sustained dysbiosis or altered microbiota composition over time [84].

Stressful events are associated with changes in the gut microbiota profile and bacterial composition, potentially impacting the microbiota-gut-brain axis [85]. Stress activates the hypothalamic-pituitary-adrenal (HPA) axis, leading to the release of cortisol. Elevated cortisol levels can alter gut physiology by increasing gut permeability, changing digestive enzymes and mucus secretion, and disrupting the gut microbiota [86]. Various forms of stress, including psychosocial stress, chronic stress, naturalistic stressors, and early life stress (such as childhood traumas and in-utero stress), can induce dysbiosis in both animals and humans [87]. Stressful events in frontline healthcare workers (FHWs) have been associated with gut dysbiosis. For instance, during the COVID-19 pandemic, FHWs experienced gut dysbiosis for around half a year which has been linked to *Faecalibaterium* and *Eubacterium eligens* [88]. In contrast, exercise has been shown to regulate gut microbiota, increase colonic epithelial growth, and enhance gut barrier [89].

### Dysbiosis and Colon Cancer

The development of CRC is a complex, multistep process that progresses from normal epithelial cells to adenomas and ultimately to carcinoma [90]. Dysbiosis has been shown to influence several stages of CRC development [91]. Specific changes in the microbiota, such as an overgrowth of pro-inflammatory bacteria, can alter the colonic environment and drive chronic inflammation, which in turn promotes cellular mutations and uncontrolled cell proliferation [16]. Microbial metabolites, particularly short-chain fatty acids (SCFAs), play a critical role in regulating immune responses and maintaining gut epithelial integrity. However, an imbalance in the microbiome can reduce the production of beneficial metabolites and increase harmful metabolites that can promote carcinogenesis [92]. For instance, some bacteria produce bile acid metabolites that can damage the DNA of colonocytes, leading to mutations and tumorigenesis [93]. Additionally, microbial-induced inflammation can alter gene expression and trigger the activation of inflammatory pathways that support cancer progression [94].

**Fig. 2:**
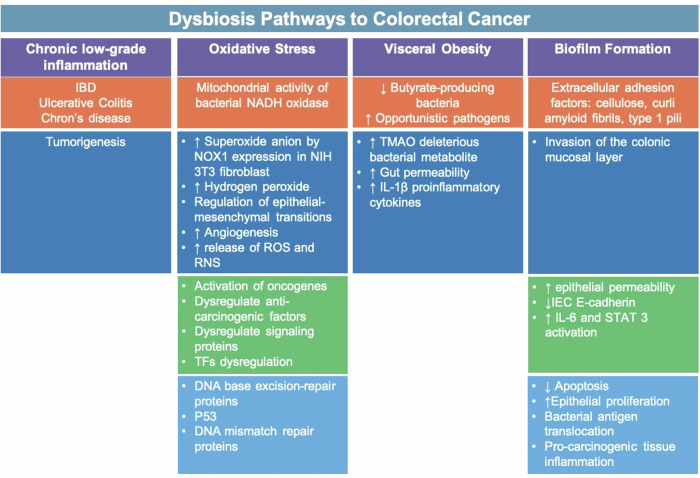
Dysbiosis Pathways to Colorectal Cancer. Chronic low-grade inflammation caused by inflammatory bowel disease (IBD) such as ulcerative colitis and Crohn’s disease can contribute to tumorigenesis and Oxidative Stress caused by increased bacterial activity of mitochondria NADPH oxidases can lead to increased superoxide anion by the expression of NOX1 in NIH 3T3 fibroblasts, increased hydrogen peroxide, regulation of epithelial-mesenchymal transitions, and increased angiogenesis. The increase in reactive oxygen species (ROS) activates oncogenes and dysregulates anti-carcinogenic factors (DNA base excision-repair proteins, p53, and DNA mismatch repair proteins), signaling proteins, and transcription factors. Visceral Obesity is associated with decreased Butyrate-producing bacteria and increased opportunistic pathogens. This leads to increased TMAO deleterious bacterial metabolite, gut permeability, and IL-1β proinflammatory cytokines. Biofilm Formation which consists of extracellular adhesion factors (cellulose, curli amyloid fibrils, and type 1 pili) invade the colonic mucosal layer. This causes increased epithelial permeability, decreased IEC E-cadherin, and increased activation of IL-6 and STAT3 which subsequently leads to decreased apoptosis and increased epithelial proliferation. This enables bacterial antigen translocation and expands pro-carcinogenic tissue inflammation

While the microbiota maintains a normal physiological GI environment, it can induce the progression of CRC in certain pathophysiological states; mainly dysbiosis (Fig. 2) [95]. Dysbiosis can contribute to colorectal cancer progression in the presence of pathogenic and cancer-inducing bacteria such as *Fusobacterium nucleatum* and *Bacteroides fragilis* [96]. These bacteria and others promote carcinogenic processes such as cell proliferation, angiogenesis, and loss of apoptosis [97, 98]. While the exact mechanisms are not fully understood, several pathways have been hypothesized including the induction of chronic inflammation, DNA damage, and production of carcinogenic metabolites [99, 100]. Chronic inflammation is a widely established risk factor for CRC [100]. Patients having IBD, including Crohn’s disease or ulcerative colitis, are susceptible to developing CRC [101]. Gut microbiota interacts with the immune system and constant stimulation of the immune system causes chronic low-grade inflammation that can progress to tumorigenesis. CRC can be caused by inflammation induced by the microbiota or bacteria-derived products [102]. For instance, *Fusobacterium nucleatum* has been shown to induce inflammation through its interaction with Toll-like receptors and contribution to DNA damage [103].

In addition, oxidative stress induced by microbiota can contribute to the development of CRC [104]. Oxidative stress is a pro-oxidative state caused by increased reactive oxygen species (ROS) and reactive nitrogen species (RNS) [105]. Both commensal and pathogenic bacteria can promote ROS release by altering mitochondrial activity or activating NADPH oxidases. The balanced production of ROS by bacterial microbiota makes these microorganisms key players in intestinal redox equilibrium [106]. In cases of intestinal stress, the pro-oxidative molecules are upregulated and increase the risk of CRC. These states of stress are mainly attributed to inflammation and DNA damage [107]. The pro-oxidative molecules activate oncogenes or dysregulate anti-carcinogenic factors (e.g. DNA base excision-repair proteins, p53, and DNA mismatch repair proteins), signaling proteins, or transcription factors [108]. Furthermore, ROS species influence the cellular proliferation that occurs in tumorigenesis. For instance, increased superoxide anion release is induced by the expression of *NOX1* in NIH 3T3 fibroblasts, causing a tenfold increase in hydrogen peroxide release [107]. Oxidative stress promotes pathways involved in the progression of epithelial-mesenchymal transitions which constitutes a critical part of the tumorigenesis cascade [109]. Similarly, CRC-related angiogenesis is highly dependent on ROS levels. CRC cells have increased expression of HIF-1, a regulator of O2 homeostasis, and are activated by oxygen-dependent mechanisms and vascular endothelial growth factor A [110].

Obesity is another microbiota-associated CRC risk factor. Obesity is known to have a strong association with non-communicable diseases like cancer [111]. In colorectal cancer (CRC), visceral obesity is associated with a 1.2 to 2-fold increased risk of cancer development [112]. Obesity in CRC patients has been associated with decreased butyrate-producing bacteria and increased opportunistic pathogens [113]. Biofilm formation by gut microbiota bacteria could promote CRC progression [114]. Biofilms are aggregations of endogenous bacteria from the host’s microbiota enclosed within a polymeric matrix. This matrix is composed of extracellular adhesion factors including cellulose, curli amyloid fibrils, and type 1 pili [115]. Biofilms invade the colonic mucosal layer to reach mucosal epithelial cells [101]. This leads to increased epithelial permeability, decreased IEC E-cadherin, and increased activation of IL-6 and STAT3. Subsequently, CRC progression is stimulated through decreased apoptosis and increased epithelial proliferation. Microbial biofilm formation can increase epithelial permeability to enable bacterial antigen translocation and expand pro-carcinogenic tissue inflammation [116]. Additionally, biofilms can influence the local immune response and contribute to antibiotic resistance [117].

The gut microbiome interacts closely with the immune system to maintain intestinal homeostasis. The immune system is responsible for distinguishing between beneficial and harmful microbes, ensuring that inflammation occurs only when necessary [118]. Regulatory T cells (Tregs) and other immune cells in the gut contribute to immune tolerance, preventing an overactive immune response to the microbiota [119]. However, dysbiosis can disrupt this balance, leading to chronic low-grade inflammation that may contribute to the development of CRC. Inflammatory cytokines, such as TNF-α and IL-6, are frequently elevated in the presence of dysbiosis and can promote tumorigenesis by stimulating cell proliferation and inhibiting apoptosis [120]. Furthermore, changes in the gut microbiota can influence the composition and activity of immune cells, such as macrophages and dendritic cells, which play a role in tumor immunity and response to treatment [121].

### Dysbiosis and Related Biomarkers

Key biomarkers of dysbiosis include Microbial Diversity Indices, Specific Microbial Species, and some Metabolites. The diversity of microbiota is represented by an alpha diversity which indicates the number and evenness of microbial species present in the gut. It is often associated with conditions such as IBD and obesity [122]. Moreover, overrepresentation of pathogenic bacteria like *Escherichia coli* or depletion of beneficial species like *Faecalibacterium prausnitzii* serves as a diagnostic marker [123]. Changes in short-chain fatty acids (SCFAs), bile acids, and tryptophan metabolism products are significant biomarkers of microbial activity and function [124]. Gut microbiota dysbiosis has been related to biomarkers for the screening and prognosis of CRC treatment [101]. Diverse study methods have been employed to detect possible CRC biomarkers including 16S rRNA sequencing, digital PCR, and qPCR [125, 126]. The 16rRNA sequencing is useful for identifying broad bacterial community compositions, while digital PCR and qPCR are more quantitative and specific [127]. Colorectal adenomas are recognized precursors to CRC, classified as precancerous lesions that can develop into CRC if not removed [101]. Therefore, they serve as effective screening markers for high-risk patients [101].

Specific changes in the microbiota balance have been linked to distinct stages of CRC [128]. Bacteria associated with an increased risk of CRC were identified in tissue biopsies and fecal samples of CRC individuals. For instance, *Fusobacterium* is widely present in CRC patients and its fecal quantification is an important biomarker. In addition, combining fecal immunochemical tests with CRC-inducing bacteria screening increases the detection rate of CRC [129]. Similarly, through sequencing 16S rRNA genes from stool samples of 490 patients, CRC was found to be linked to the following bacteria: *Peptostreptococcus stomatis, Porphyromonas assaccharolytica, Parvimonas micra*, and *Fusobacterium nucleatem*.

Recent advancements in biomarker-based therapies have revolutionized the field of dysbiosis treatment, emphasizing personalized and targeted approaches. Microbiome-based precision medicine has introduced tailored probiotics and prebiotics designed to restore microbial balance, alongside refined fecal microbiota transplantation (FMT) techniques [130, 131]. Gene-editing technologies, particularly CRISPR-Cas systems, enable precise modulation of microbial gene expression, targeting pathogenic traits within the microbiota [132]. Additionally, biomarker-guided cell therapies including engineered regulatory T cells for immune modulation and synthetic microbial strains can produce therapeutic metabolites or modulating immune responses based on specific biomarkers. These innovations highlight the potential of biomarkers in bridging diagnostics and therapeutic strategies, paving the way for the integration of precision medicine in managing dysbiosis-related conditions [133].

## Conclusion and Future Perspectives

This review highlights the relationship between the gut microbiota and colorectal health, with a focus on colon barrier structure and function, immune system interactions, and microbiota-based interventions. The gut microbiota plays a key role in maintaining colon barrier integrity and modulating immune responses, both of which are critical in preventing disease. Dysbiosis, characterized by an imbalance in microbial composition, has been linked to disruptions in these systems and an increased risk of colon cancer. Factors contributing to dysbiosis include diet, lifestyle, and environmental influences, all of which can alter the microbiota and its metabolites. Additionally, dysbiosis-associated biomarkers show promise for diagnostic and therapeutic applications but require further validation. Beyond CRC, gut microbiota imbalances have also been implicated in other colon diseases, such as inflammatory bowel disease and diverticulitis, suggesting shared microbial pathways. Understanding these connections could lead to more effective prevention and treatment strategies. Although extensive research has been conducted to identify the relationship between the gut microbiome and CRC, the exact relationship remains unclear [134]. While there is evidence of an association between gut microbiota composition and CRC, establishing causation remains challenging. It is yet to be proved whether alterations in the microbiota contribute to CRC development, or if changes in the gut environment post-CRC led to changes in the gut microbiome [135]. Furthermore, the role of specific bacteria species or strains and their functional activities in CRC remains not well identified. The exact mechanisms by which all the gut bacteria or microbial metabolites influence CRC progression are also yet to be determined [136]. While advancements in microbiota-based therapies, such as probiotics, prebiotics, and fecal microbiota transplantation, are promising, clinical applications remain in their early stages.

Longitudinal prospective and large international cohorts are needed to validate and further expand the available research data on these pathways [137]. While there is rising interest in using the gut microbiota as a diagnostic or therapeutic target for CRC, clinical applications are still in the early stages and further studies are needed to clarify their potential benefits and limitations [138].

## Data Availability

No datasets were generated or analysed during the current study.
